# Risk Factors for Intolerable Postoperative Pain After Vitreoretinal Surgery Under AoA-Guided General Anesthesia with Intravenous COX-3 Inhibitors: A Post Hoc Analysis

**DOI:** 10.3390/ph18121826

**Published:** 2025-12-01

**Authors:** Michał J. Stasiowski, Kaja Marczak, Anita Lyssek-Boroń, Nikola Zmarzły

**Affiliations:** 1 Chair and Department of Emergency Medicine, Faculty of Medical Sciences in Zabrze, Medical University of Silesia, 40-055 Katowice, Poland; 2 Department of Anaesthesiology and Intensive Care, 5th Regional Hospital, Trauma Centre, 41-200 Sosnowiec, Poland; kaja.marczak.wss5@gmail.com; 3 Department of Ophthalmology with Paediatric Unit, 5th Regional Hospital, Trauma Centre, 41-200 Sosnowiec, Poland; anitaboron3@gmail.com; 4 Department of Ophthalmology, Faculty of Medicine, Academy of Silesia, 40-055 Katowice, Poland; 5 Collegium Medicum, WSB University, 41-300 Dabrowa Gornicza, Poland; nikola.zmarzly@wsb.edu.pl

**Keywords:** vitreoretinal surgery, general anesthesia, paracetamol, metamizole, adequacy of anesthesia, intolerable postoperative pain perception, numeric pain rating scale

## Abstract

**Background/Objectives**: Intolerable postoperative pain perception (IPPP) may occur in patients undergoing vitreoretinal surgery (VRS), while general anesthesia (GA) is often preferred over regional techniques due to multiple contraindications. Intraoperative administration of intravenous rescue opioid analgesics (IROA) during GA increases the risk of perioperative adverse events; however, this requirement can be reduced through preventive analgesia. The Adequacy of Anesthesia (AoA) concept, based on entropy EEG and the Surgical Pleth Index (SPI), allows real-time titration of IROA to maintain optimal nociception/anti-nociception balance and create comparable intraoperative conditions across patients. This study aimed to identify risk factors for IPPP after VRS performed under AoA-guided GA combined with intravenous preventive analgesia using COX-3 inhibitors. **Methods**: A total of 165 patients scheduled for VRS were randomized to receive AoA-guided GA combined with intravenous preventive analgesia using either paracetamol plus metamizole, paracetamol alone, or metamizole alone. **Results**: Data from 153 patients were analyzed. Neither age, body mass index, smoking status, arterial hypertension, diabetes mellitus, intraoperative noxious maneuvers, demand for IROA, nor length of surgery correlated with the incidence of IPPP under AoA-guided GA. The combination of paracetamol and metamizole resulted in the lowest rate of IPPP among all groups. **Conclusions**: AoA-guided GA combined with COX-3 inhibitors appears to standardize intraoperative nociception/anti-nociception balance in patients undergoing VRS, effectively mitigating most known risk factors for IPPP, with female sex independently associated with its occurrence. We recommend the optimization of perioperative pharmacotherapy through individualized AoA-guided GA with intravenous COX-3 inhibitors to minimize IPPP incidence.

## 1. Introduction

Intolerable postoperative pain perception (IPPP), although a subjective phenomenon and difficult to quantify, markedly deteriorates postoperative outcomes, impairs cost-effectiveness, and decreases patient satisfaction with medical care [1]. The prevalence of IPPP, regardless of the type of surgery, is estimated to range between 30% and 75% [2,3]. Therefore, identifying risk factors associated with IPPP and raising awareness among healthcare professionals to implement preventive measures are of great importance [4]. Several preoperative predictors of poor postoperative pain control have been reported, including younger age, female sex, history of depression, preoperative anxiety, sleep difficulties, higher body mass index, presence of preoperative pain, and preoperative use of analgesics [5].

The prevalence of IPPP following ophthalmic surgeries is often underestimated and consequently neglected [6], mainly due to limited understanding of the specific characteristics of these procedures, leading to inadequate postoperative pain management [7]. The reported incidence of severe pain after ophthalmic surgery ranges from 13.8% in procedures such as cataract extraction, iridectomy, and anterior chamber revision, to as high as 53.8% after encircling band placement or strabismus surgery [8].

Given the multifactorial nature of postoperative pain in ophthalmic surgery, further research is warranted to explore anesthetic regimens that effectively prevent IPPP and facilitate smoother recovery [9]. Prevention of IPPP relies on appropriate preventive analgesia, adequate intraoperative anti-nociception, and effective postoperative pain relief, which is considered a fundamental human right [10]. Preventive analgesia reduces the demand for intraoperative rescue opioid analgesia (IROA), improves the intraoperative nociception/anti-nociception balance, and promotes smoother postoperative recovery with decreased need for postoperative analgesia [5].

Intravenous administration of metamizole or paracetamol, two widely used cyclooxygenase-3 (COX-3) inhibitors [11,12,13], has demonstrated efficacy in postoperative pain control, even after a single dose, with a favorable safety profile [14,15,16,17,18,19,20]. Compared with standard nonsteroidal anti-inflammatory drugs (NSAIDs), these agents are particularly suitable for patients at increased risk of gastrointestinal or renal complications, providing safety for the upper gastrointestinal tract and kidneys in cases where NSAIDs are contraindicated [21,22].

Proper nociception/anti-nociception balance can also be achieved through digital intraoperative monitoring, which is gaining popularity [23]. Commonly used methods include pupillometry, adequacy of anesthesia (AoA) monitoring, and the nociception level index, depending on the type of surgery [24]. Use of AoA-guided general anesthesia (GA) has been associated with fewer intraoperative adverse events, reduced IROA consumption, improved hemodynamic stability, shorter emergence from GA, and lower incidence of IPPP. This approach involves titration of IROA based on fluctuations in the Surgical Pleth Index (SPI) and adjustment of the hypnotic component of GA according to entropy EEG values, including response entropy (RE) and state entropy (SE) [24,25,26,27,28,29,30,31].

Encouraged by previous reports emphasizing the importance of early identification of IPPP predictors to enhance postoperative recovery, the present post hoc analysis was conducted to identify risk factors for IPPP after vitreoretinal surgery (VRS) under AoA-guided GA combined with intravenous COX-3 inhibitor-based preventive analgesia. Unlike previous publications from this cohort, this study specifically focuses on risk factor identification, providing new insights to guide preventive strategies in VRS.

## 2. Results

Of the 165 patients initially enrolled, nine were excluded due to inability to report postoperative pain, technical issues with SPI monitoring, or postoperative agitation preventing observation during Stage 5. Consequently, data from 153 patients were included in the final analysis (Figure S1). As demonstrated in our previous publications based on the same study cohort [32,33], significant intergroup differences in sex distribution, height, weight, and BMI were observed. Detailed demographic data are provided in Supplementary Table S1.

Overall, 16 patients (10.5%) reported IPPP: 9 (18%) patients in the M group, 6 (12%) in the P group, and 1 (2%) in the PM group (Table 1). In the multivariable logistic regression including sex and age, female sex was the only factor significantly associated with IPPP (men had significantly lower odds of IPPP: OR 0.22, 95% CI 0.05–0.78, *p* = 0.03). For NPRS max, neither sex nor age acted as confounders. Regarding intraoperative FNT demand, the M group required significantly more rescue doses than the P group. Additionally, age >65 years was identified as a confounding factor for both intraoperative FNT administration (95% CI: −79.2 to −13.8, *p* = 0.004) and intraoperative fluid management (95% CI: −266.4 to −44.6, *p* = 0.009).

Consequently, an additional multivariable analysis was performed to explore the relationship between mean IROA dose, duration of surgery, and intraoperative SPI values (mean, maximum, and minimum) in patients reporting tolerable (NPRS ≤ 3) or intolerable (NPRS > 3) pain, regardless of group allocation (Table 2).

Age > 65 years was identified as a confounding factor for IROA dose, with older patients receiving lower doses (*p* = 0.004), and for duration of surgery, which was shorter in older patients (*p* = 0.01). Sex was not a significant confounder. Patients with intolerable pain had significantly higher mean, maximum, and minimum SPI values compared with those reporting tolerable pain, while neither sex nor age significantly influenced intraoperative SPI values.

No significant associations were observed between age, BMI, hypertension, diabetes mellitus, smoking status, or most noxious intraoperative maneuvers and the incidence of intolerable postoperative pain (NPRS > 3). A significant association was observed only for sex, with women being overrepresented among patients reporting intolerable postoperative pain (Table 3).

## 3. Discussion

The primary aim of this study was to investigate the optimization of perioperative pharmacotherapy using both paracetamol and metamizole, compared with their single use, in patients undergoing VRS under digital monitoring with AoA guidance to titrate IROA. Combined preventive analgesia with these two COX-3 inhibitors significantly reduced the incidence of IPPP compared with monotherapy, which was the primary outcome of the previous study [32]. In addition, no advantage was achieved in terms of the incidence of oculocardiac reflex (OCR), perioperative nausea and vomiting (PONV), and oculoemetic reflex, although the overall rate of PONV was low [33].

In this cohort, IPPP occurred in 16 of 153 patients (10.5%), and only 1 of 53 patients (1.88%) receiving combined preventive analgesia experienced IPPP. This prompted a post hoc analysis to identify potential risk factors for IPPP and elucidate potential mechanisms underlying this adverse event, and providing new insight into patient-specific factors beyond the primary outcome. Despite the low overall incidence, likely due to the multimodal approach combining intravenous preventive analgesia with intraoperative opioid titration under AoA monitoring, identifying individual risk factors remains essential for personalized perioperative management. Multivariable logistic regression confirmed that female sex was the only confounding factor for IPPP, suggesting that, for most patients, the combined preventive analgesia and AoA-guided opioid titration effectively mitigated other potential risk factors. Moreover, patients experiencing IPPP exhibited significantly higher mean, maximum, and minimum SPI values, highlighting the association between intraoperative nociception and postoperative pain intensity, independent of sex and age.

Pharmacologically, preoperative administration of preventive analgesia reduces the requirement for intraoperative opioids and lowers the risk of PONV [15,20], while providing effective postoperative analgesia even after a single dose, lasting several hours and ensuring effective analgesia throughout VRS and the postoperative period in the Post-Anesthesia Care Unit, until oral medication can be safely offered [19]. Paracetamol and metamizole exert both central (COX-3 inhibition) [34] and peripheral effects. Paracetamol acts via multiple pathways [35,36], including the endogenous opioid pathway [37], activation of 5HT1A/B receptors in the bulbospinal serotonergic pathway [38], involvement in the nitric oxide pathway [39], and enhancement of cannabinoid/vanilloid signaling through CB1 receptors [40,41,42]. Metamizole primarily reduces inflammatory pain by inhibiting prostaglandin synthesis, while also providing analgesic, antipyretic, and anti-inflammatory effects [43].

Even with preventive analgesia, IPPP may still occur. Recent studies have focused on digital monitoring of GA to improve outcomes [24], as volatile anesthetics tend to blunt intraoperative hemodynamic responses in the presence of insufficient anti-nociception [44]. AoA-guided GA combines entropy EEG (RE and SE) for depth of hypnosis with SPI monitoring for nociception, ranging from 0 (no nociception) to 100 (maximum nociception). Efficient titration of IROA based on SPI during noxious maneuvers reduces the risk of IPPP [26,27].

Risk factors for IPPP vary by surgery type [45]. Posterior segment, corneal, and extraocular muscle surgeries are associated with higher postoperative pain intensity. Anterior segment surgery, which typically causes less pain, may nevertheless result in more intense pain perception when performed under GA compared with regional anesthesia techniques [8]. In glaucoma surgery, preoperative anxiety, cyclophotocoagulation, and phacotrabeculectomy, combined with or without intraocular lens implantation, have been reported as significant predictors of early postoperative pain [46]. In patients undergoing phacovitrectomy, IPPP was reported more frequently than in patients undergoing PPV alone [47], which was not observed in the present cohort.

In our recent analyses, paracetamol effectively maintained the nociception/anti-nociception balance, as reflected by SPI values during the most noxious intraoperative maneuvers such as endolaser treatment, indentation, and vitrectomy-related maneuvers, showing comparable efficacy to peribulbar block using a 1:1 mixture of 2% lidocaine with 0.5% bupivacaine, and similar effectiveness to regional anesthesia during trocar insertion [48]. Paracetamol alone was as effective as peribulbar block regardless of the anesthetic mixture [49]. These findings support the current observation that combined preventive analgesia with paracetamol and metamizole provides low rates of IPPP compared with metamizole alone, with no significant difference compared with paracetamol alone.

Before the introduction of digital monitoring and widespread clinical adoption, numerous risk factors for IPPP were identified to personalize GA and minimize postoperative pain. According to Lesin et al., independent predictors of mean postoperative pain intensity included omission of premedication, surgery under GA, higher preoperative pain intensity, and pain catastrophizing. Predictors of postoperative pain duration included preoperative pain intensity, type of anesthesia, and self-assessed health status [6]. Overall, preoperative pain, anxiety, age, and type of surgery were considered the four most significant predictors of IPPP, two of which were not relevant in the present cohort [50]. In contrast, female sex emerged as a confounding factor potentially influencing IPPP incidence, consistent with previous reports highlighting sex as a risk factor [51], alongside younger age, depressive symptoms, anxiety, sleep difficulties, higher body mass index, and preoperative analgesic use [52].

Presumably, the increased sympathetic tone characteristic of female sex before anesthesia and surgery may predispose these patients to increased pain sensitivity, potentially reflected in increased SPI values prior to induction of GA, correlating with IPPP [53]. Current literature also reports the feasibility of observing SPI values during recovery from GA to predict postoperative moderate to acute pain (NPRS > 3) [54]. Early identification of at-risk patients may enable tailored interventions, although the exact mechanisms remain to be fully elucidated. Interestingly, comorbidities such as diabetes and hypertension were not associated with postoperative pain intensity in this study, contrasting with prior reports [46].

In our opinion, further studies could explore non-pharmacological modalities to reduce preoperative anxiety and pain sensitivity in female patients undergoing AoA-guided VRS with preventive analgesia using paracetamol and metamizole, who unfortunately report IPPP. Interventions such as music therapy, aromatherapy, and acupuncture have demonstrated efficacy in reducing preoperative anxiety and postoperative pain in women undergoing breast cancer surgery [55]. Dedicated anesthetic planning, along with preoperative anxiety assessment using validated tools [56], in a supportive environment free from workload constraints, may further improve outcomes [57].

Several limitations should be considered. First, the short duration of emergence from GA limited the number of hemodynamic measurements in some cases. Second, postoperative pain perception might have been influenced by volatile anesthetics, IROA, and preoperative sedatives. Third, pain is inherently subjective and difficult to quantify. Fourth, this post hoc analysis relies on the same cohort as previously published primary and secondary outcomes. Finally, a protocol using ΔSPI > 15 as the threshold for IROA was adopted to avoid potential opioid overdose, although stricter thresholds (ΔSPI > 10 or SPI > 50) have been used in other studies [58,59,60].

## 4. Materials and Methods

### 4.1. Patients

The study protocol adhered to the principles of the Declaration of Helsinki and was approved by the Bioethics Committee of the Medical University of Silesia (approval number KNW/0022/KB1/122/17, issued on 5 December 2017; Chair: PhD, MD Bogusław Okopień), prior to data collection. The prospective, randomized trial was registered in the Clinical Trials Registry (NCT03389243, Silesian MUKOAiIT7, 27 December 2017). Data collection took place between 15 January 2018 and 16 December 2022. The study was initially approved for a 3-year period; however, due to the SARS-CoV-2 pandemic, nationwide lockdowns, and temporary suspension of elective surgeries, these periods were officially excluded from the approved timeframe. Consequently, the entire study duration remained covered by valid ethical approval and institutional oversight. All data used in this post hoc analysis were collected under full ethical supervision and validated to ensure integrity.

Inclusion criteria comprised: no history of increased intraocular pressure, absence of previous ocular surgery, and no prior treatment with antiglaucoma medications. Exclusion criteria included any condition affecting posterior segment anatomy or function predisposing to glaucoma (uveitis, penetrating ocular trauma, retinal vein occlusion, scleral buckling), pregnancy, alcohol or drug abuse, neurological disease or neurosurgical procedures that could interfere with entropy EEG, pulmonary disease, abuse or allergy to metamizole or paracetamol, presence of chronic pain or acute pain, predicted difficult placement of laryngeal mask, and cardiac arrhythmia affecting SPI monitoring.

A total of 165 patients (ASA I–III) provided written informed consent and were enrolled. Randomization was conducted using sealed envelopes to ensure allocation concealment.

Patients were randomly assigned to one of three groups. Group M received GA with a single intravenous dose of metamizole 2.5 g (Polpharma S.A., Gdańsk, Poland) in 100 mL saline, administered 30 min before transfer to the operating room. Group P received GA with a single intravenous dose of acetaminophen 1 g (Fresenius Kabi, Błonie, Poland) in 100 mL saline, administered 30 min before transfer to the operating room. Group PM received GA with both preventive analgesic regimens described above, in accordance with the most recent guidelines for postoperative pain management [61].

During the preoperative visit, patients were instructed in the Numeric Pain Rating Scale (NPRS, 0–10), where 0 indicated no pain and 10 represented the worst imaginable pain.

### 4.2. Anesthetic Protocol

All patients fasted for at least 12 h before surgery. On the day of surgery, each patient received intravenous midazolam (3.75–7.5 mg, Roche, Warsaw, Poland) according to body weight and age [62]. Preoxygenation with 100% oxygen was performed for ~5 min, and 10 mL/kg Ringer solution was infused.

Anesthesia induction was performed with intravenous fentanyl (FNT) at 1 µg/kg and propofol at 2.5 mg/kg (Fresenius, Cairo, Egypt). After loss of consciousness, all patients received rocuronium at 0.6 mg/kg (Fresenius, Błonie, Poland), followed 45 s later by laryngeal mask insertion. End-tidal carbon dioxide was maintained between 35 and 37 mmHg. Before surgery began, sevoflurane (Sevorane, Baxter Healthcare Ltd., Thetford, UK) concentration was adjusted to maintain a SE value of approximately 40–45 throughout the surgery.

Throughout induction and surgery, standard monitoring was employed, including heart rate, non-invasive arterial pressure, arterial blood saturation, standard electrocardiography, fraction of inspired oxygen, fraction of inspired and expired sevoflurane, end-tidal carbon dioxide, minimal alveolar concentration of sevoflurane. Depth of anesthesia was assessed with the entropy EEG (SE and RE), intraoperative analgesia was guided by the SPI, and muscle relaxation was also monitored (Carescape B650, GE Healthcare, Helsinki, Finland).

#### 4.2.1. Stage 1

Upon admission to the operating theater, sensors for EEG (SE, RE), SPI, blood pressure, and ECG were applied according to manufacturer instructions. Baseline values were recorded.

#### 4.2.2. Stage 2

Mean SPI values were recorded beginning 5 min after laryngeal mask insertion until the start of orbital sterilization, allowing for SPI sensor calibration.

#### 4.2.3. Stage 3: Intraoperative

SPI was continuously recorded every minute. If SPI increased by more than 15 points (ΔSPI > 15) above Stage 2 mean, a rescue dose of FNT 1 µg/kg was administered every 5 min until SPI returned to baseline. The duration of VRS was measured from speculum insertion to removal. The initial FNT dose (1 µg/kg) was assumed to provide sufficient analgesia before speculum placement.

According to Gruenewald et al. (2013) [24], ΔSPI > 10 or an absolute SPI value > 50 predicts suboptimal analgesia, while other studies have used SPI > 50 alone as the threshold [23]. In this study, a threshold of ΔSPI > 15 for at least one minute was adopted to reduce the risk of FNT overdosage due to transient SPI variability.

All surgeries were performed by the same experienced ophthalmic surgeon (A.L-B., >400 procedures/year).

Standard 23-gauge vitrectomy was performed with three or four ports, followed by core and peripheral vitrectomy with scleral indentation. Epiretinal and/or internal limiting membrane peeling was performed as indicated. If necessary, four types of tamponades were applied: perfluorocarbon liquids, air, sulfur hexafluoride, silicone oil.

Retinal breaks or degenerations were treated using laser photocoagulation. Occurrence of OCR (≥20% decrease in heart rate during ocular manipulation) was documented, and atropine 0.5 mg was administered intravenously if bradycardia persisted.

#### 4.2.4. Stage 4: Emergence from GA

During emergence, patients were further monitored by the anesthesiology team.

#### 4.2.5. Stage 5: Postoperative Period

In the Post-Anesthesia Care Unit, patients were further monitored by the anesthesiology team blinded to group allocation. Adverse events, including PONV, sedation level, and allergic reactions, were recorded along with pain intensity during the first 24 h. PONV was treated with ondansetron 4 mg intravenously (Accord Healthcare Limited, Harrow, UK). If mean arterial pressure fell below 65 mmHg, 5 mL/kg Optilyte solution was infused. Oxygen was administered at 3 L/min via nasal cannula.

Pain intensity was assessed every 10 min using the NPRS (0–10). It was categorized as mild (0–3), moderate (4–6), or severe (7–10). When NPRS exceeded 3, patients received standard NSAID analgesia, either dexketoprofen (Berlin-Chemie Menarini, Berlin, Germany) or ibuprofen (B. Braun, Melsungen, Germany), in addition to the assigned preventive analgesic, according to current guidelines of the Polish Society of Anaesthesiologists [61].

Patients were observed in the Post-Anesthesia Care Unit for at least 30 min before transfer to the Department of Ophthalmology.

### 4.3. Statistical Analysis

Sample size was calculated using G*Power software (ver. 3.1.9.7; Heinrich Heine University, Düsseldorf, Germany) [63]. One-way ANOVA for three groups determined that a total of 159 patients were required. A power of 0.79 was obtained for the 153 patients finally analyzed.

Statistical analysis was performed with STATISTICA 13.3 (StatSoft, Kraków, Poland) and R (ver. 4.4.0) [64]. Normality was assessed using the Shapiro–Wilk test. Then the Kruskal–Wallis test with Dunn’s post hoc test was used for multi-group comparisons of continuous variables. The Mann–Whitney U test was applied for pairwise comparisons. Nominal variables were compared with the chi-square or Fisher’s exact test (when expected frequencies < 5). Bonferroni correction was applied for multiple testing. To account for potential imbalances between groups in sex and age, multivariable regression analyses were performed. Logistic regression was used for the binary outcome of IPPP, whereas linear regression was applied for continuous outcomes. Post hoc pairwise comparisons between groups were performed using estimated marginal means with Tukey adjustment. A *p*-value < 0.05 was considered statistically significant.

## 5. Conclusions

The combined use of COX-3 inhibitor–based preventive analgesia and AoA-guided general anesthesia offered robust perioperative analgesic control in VRS, mitigating the influence of established risk factors for IPPP. Female sex remained independently associated with higher pain perception, suggesting that additional individualized interventions may be beneficial for this subgroup. Further prospective research is needed to confirm this association and to refine personalized approaches aimed at fully preventing IPPP.

## Figures and Tables

**Table 1:** Postoperative pain assessment and intraoperative management parameters.

Scale		Total *N* = 153 (100%)	PM *n* = 52 (34%)	P *n* = 52 (34%)	M *n* = 50 (33%)	*p*-Value
Patients with postoperative pain	acute—NPRS > 6 *N* (%)	2 (1%)	1 (2%)	1 (2%)	0 (0%)	PM vs. M, *p* = 0.02
	moderate—NPRS 4–6 *N* (%)	14 (9%)	0 (0%)	5 (10%)	9 (18%)	
	mild—NPRS ≤ 3 *N* (%)	137 (89.5%)	51 (98%)	45 (88%)	41 (82%)	
	intolerable—NPRS > 3 *N* (%)	16 (10.5%)	1 (2%)	6 (12%)	9 (18%)	
Patients unable to assess their postoperative pain *n* (%)	Yes (%)	0 (0%)	0 (0%)	0 (0%)	0 (0%)	-
NPRS max X ± SD Me (IQR)	[0 ÷ 10]	1 ± 1.8 0 (2)	0.5 ± 1.4 0 (0)	1.1 ± 1.9 0 (2)	1.3 ± 2.1 0 (3)	NS *p* = 0.1
FNT requirement X ± SD Me (IQR)	[µg]	149.8 ± 90.8 100 (100)	151.8 ± 79.7 100 (100)	116.3 ± 75.4 100 (100)	180.7 ± 104.1 200 (150)	P vs. M, *p* = 0.004
Intraoperative fluid challenge X ± SD Me (IQR)	[mL]	1042.2 ± 342.6 1000 (450)	1090.4 ± 389.4 1100 (450)	958.7 ± 258.5 1000 (250)	1069 ± 351.1 1000 (450)	NS *p* = 0.05

PM—paracetamol/metamizole; P—paracetamol; M—metamizole; NPRS—numeric pain rating scale, FNT—fentanyl, SD—standard deviation; IQR—interquartile range; NS—not statistically significant.

**Table 2:** Relationship between intraoperative parameters and postoperative pain perception.

Parameter	Total *N* = 153 (100%)	NPRS > 3 *n* = 16 (10.5%)	NPRS ≤ 3 *n* = 137 (89.5%)	*p*-Value
Dose of IROA [µg] X ± SD Me (IQR)	149.8 ± 90.8 100 (100)	123.1 ± 97.1 100 (150)	152.8 ± 90 100 (100)	NS *p* = 0.7
Time of surgery [min] X ± SD Me (IQR)	47.2 ± 18.9 44 (28)	49.1 ± 19.4 49 (26)	47 ± 18.9 44 (28)	NS *p* = 0.1
Mean SPI in stage 5 X ± SD Me (IQR)	56.4 ± 15.2 57.4 (25.4)	70.5 ± 9.8 72.1 (13)	54.7 ± 14.9 55.3 (27)	*p* < 0.001
Max SPI in stage 5 X ± SD Me (IQR)	66.8 ± 14.7 68 (23)	78.5 ± 9 79.5 (17)	65.4 ± 14.6 68 (22)	*p* < 0.001
Min SPI in stage 5 X ± SD Me (IQR)	46.8 ± 16.5 48 (29)	61.9 ± 9.7 62 (12)	45.1 ± 16.3 45 (28)	*p* < 0.001

NPRS—numeric pain rating scale, IROA—intravenous rescue opioid analgesics, SPI—surgical pleth index; SD—standard deviation; IQR—interquartile range; NS—not statistically significant.

**Table 3:** Patient- and surgery-related risk factors according to postoperative pain intensity (NPRS ≤ 3 vs. NPRS > 3), irrespective of group allocation.

**NPRS**	**Sex**			**Diabetes Mellitus**					
	**Male**	**Female**	***p*-Value**	**Insulin-Dependent**		**Insulin-Independent**	**Non-Diabetics**		***p*-Value**
≤3	68	69	*p* = 0.03	49		22	66		NS *p* = 0.3
>3	3	13		3		2	11		
**NPRS**	**Arterial hypertension**			**Weight**					
	**Yes**	**No**	***p*-Value**	**Underweight**		**Normal weight**	**Overweight/Obese**		***p*-Value**
≤3	82	38	NS *p* = 0.3	0		36	82		NS *p* = 0.5
>3	8	7		0		9	6		
**NPRS**	**Age**			**Smoking status**					
	**>65 years**	**≤65 years**	***p*-Value**	**Yes**		**No**	***p*-Value**		
≤3	58	62	NS *p* = 0.6	14		123	NS *p* = 0.5		
>3	9	6		2		14			
**Intraoperative maneuvers**									
**NPRS**	**Endolaser Treatment**			**Indentation**			**Phacovitrectomy**		
	**Yes**	**No**	***p*-Value**	**Yes**	**No**	***p*-Value**	**Yes**	**No**	***p*-Value**
≤3	110	27	NS *p* = 0.2	49	88	NS *p* = 0.2	81	56	NS *p* = 0.8
>3	10	6		9	7		10	6	

NPRS—numeric pain rating scale, NS—not statistically significant.

## Data Availability

The original contributions presented in this study are included in the article/supplementary material. Further inquiries can be directed to the corresponding author.

## Supplementary Materials

The following supporting information can be downloaded at: https://www.mdpi.com/article/10.3390/ph18121826/s1, Figure S1: Flow diagram of patient enrollment, randomization, exclusions, and final analysis for the study participants; Table S1. Anthropometric characteristics of patients in the study groups.

## Abbreviations

The following abbreviations are used in this manuscript:

AoA	adequacy of anesthesia
COX-3	cyclooxygenase-3
FNT	fentanyl
GA	general anesthesia
IPPP	intolerable postoperative pain perception
IROA	intravenous rescue opioid analgesia
M	metamizole
NPRS	numeric pain rating scale
OCR	oculocardiac reflex
P	paracetamol
PM	paracetamol/metamizole
PONV	perioperative nausea and vomiting
RE	response entropy
SE	state entropy
SPI	surgical pleth index
VRS	vitreoretinal surgery
