# Supplementary material for: Risk Factors for Intolerable Postoperative Pain After Vitreoretinal Surgery Under AoA-Guided General Anesthesia with Intravenous COX-3 Inhibitors: A Post Hoc Analysis
Source: Pharmaceuticals (Basel). 2025 Dec 1;18(12):1826. doi: 10.3390/ph18121826 (PMC12736296; doi:10.3390/ph18121826)
Supplement: Supplementary file 1 [file pharmaceuticals-18-01826-s001.zip › Table S1.pdf]

pharmaceuticals

MDPI

Table S1. Anthropometric characteristics of patients in the study groups.

Characteristics		Total<br>*N* = 153 (100%)	PM<br>*n* = 52 (34%)	P<br>*n* = 52 (34%)	M<br>*n* = 50 (33%)	*p*-Value
Age<br>X ± SD<br>Me (IQR)	[years]	63.8 ± 11.7<br>65 (12)	64.4 ± 11<br>67 (11.5)	64.6 ± 11.6<br>66 (9)	62.6 ± 12.3<br>64 (14)	NS<br>*p* = 0.7
	> 65 years	72.2 ± 4.8<br>72 (7)	71.1 ± 4.6<br>70 (6)	72.5 ± 5.4<br>70 (8)	72.9 ± 4.3<br>72 (5)	NS<br>*p* = 0.3
	≤ 65 years	55.5 ± 10.4<br>60 (14.5)	55.1 ± 10.6<br>60 (15)	56.3 ± 10.5<br>60.5 (14.5)	55.1 ± 10.7<br>60 (14)	
Gender<br>N (%)	female	82 (54%)	23 (44%)	35 (69%)	24 (48%)	PM vs. P,<br>*p* = 0.02
	male	71 (46%)	29 (56%)	16 (31%)	26 (52%)	
Height<br>X ± SD<br>Me (IQR)	[cm]	167.1 ± 9.2<br>165 (15)	169.5 ± 9<br>167 (12)	164 ± 8.3<br>164 (11)	168.3 ± 9.5<br>170 (15)	PM vs. P,<br>*p* = 0.02
Weight<br>X ± SD<br>Me (IQR)	[kg]	77.2 ± 15.4<br>77 (21)	84.97 ± 15.6<br>82 (27)	73.4 ± 14.8<br>72.5 (21.5)	74.6 ± 13.9<br>74.5 (19)	PM vs. P,<br>*p* = 0.004;<br>PM vs. M,<br>*p* = 0.009
BMI<br>X ± SD<br>Me (IQR)	[kg/m^2]	27.7 ± 5<br>26.7 (6.6)	29.6 ± 5<br>27.8 (5.9)	27.9 ± 5.3<br>27.8 (7.7)	26.3 ± 4.2<br>25.6 (4)	PM vs. M,<br>*p* = 0.007
BMI<br>*N* (%)	underweight	0 (0%)	0 (0%)	0 (0%)	0 (0%)	NS<br>*p* = 0.2
	norm	45 (29%)	8 (15%)	17 (33%)	20 (40%)	
	overweight	49 (32%)	14 (27%)	15 (29%)	20 (40%)	
	obesity	39 (25%)	15 (29%)	15 (29%)	9 (18%)	

PM—paracetamol/metamizole; P—paracetamol; M—metamizole; BMI—body mass index; SD—standard deviation; IQR—interquartile range; NS—not statistically significant.
