# Supplementary figures and images for: Risk Factors for Intolerable Postoperative Pain After Vitreoretinal Surgery Under AoA-Guided General Anesthesia with Intravenous COX-3 Inhibitors: A Post Hoc Analysis
Source: Pharmaceuticals (Basel). 2025 Dec 1;18(12):1826. doi: 10.3390/ph18121826 (PMC12736296; doi:10.3390/ph18121826)

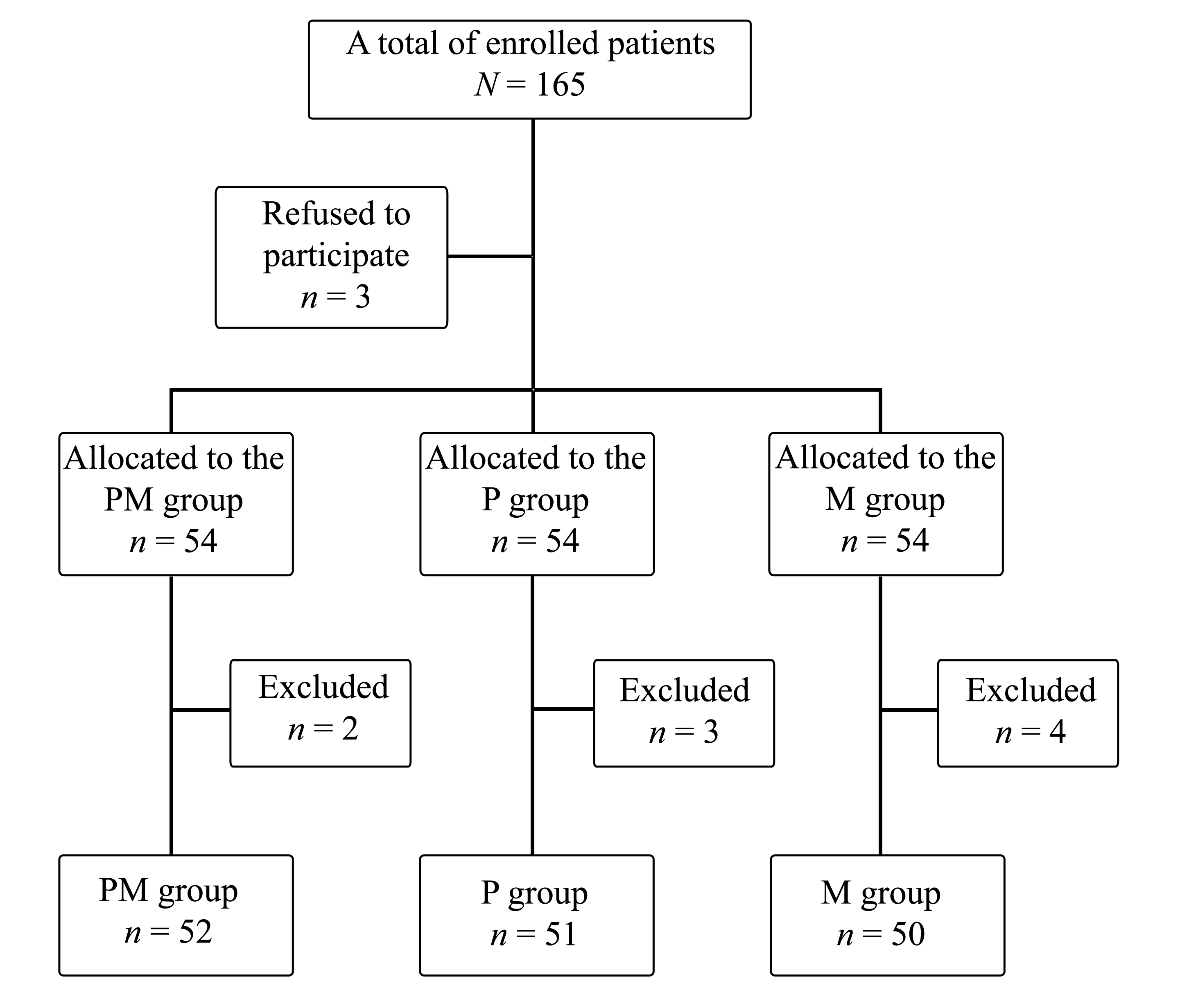

Supplement: Supplementary file 1 [file pharmaceuticals-18-01826-s001.zip › Figure S1.tiff]
